# Kyste hydatique du foie: à propos d'un mode de révélation exceptionnel

**DOI:** 10.11604/pamj.2014.18.158.2986

**Published:** 2014-06-18

**Authors:** Siham Eddeghai, Imane Eddoukani, Azzedine Diffaa, Khadija Krati

**Affiliations:** 1 Service de gastro-entérologie Centre Hospitalier Universitaire Mohammed VI Marrakech Maroc

**Keywords:** Hémorragie digestive, hypertension portale, kyste hydatique du foie, gastrointestinal bleeding, portal hypertension, hydatid cyst of the liver

## Abstract

Le kyste hydatique (KH) est une maladie parasitaire bénigne qui touche avec prédilection le foie. Il est assez fréquent dans les pays du pourtour méditerranéen, cependant il reste une cause exceptionnelle d'hypertension portale. Nous rapportons une nouvelle observation d′un patient qui présente un kyste hydatique du foie révélé par une hémorragie digestive sur un syndrome d′hypertension portale en précisant ses différentes particularités diagnostiques et thérapeutiques avec une revue de littérature.

## Introduction

Le kyste hydatique est une maladie parasitaire bénigne qui touche avec prédilection le foie. Il est assez fréquent dans les pays du pourtour méditerranéen, cependant il reste une cause exceptionnelle d'hypertension portale [1, 2, 3, 4]. Nous rapportons une nouvelle observation d'un patient qui présente un kyste hydatique du foie révélé par une hémorragie digestive par rupture de varice œsophagienne sur un syndrome d′HTP avec une revue de la littérature.

## Patient et observation

Il s'agit d'un patient âgé de 44 ans, originaire d'un milieu rural avec notion de contact avec les chiens, qui s'est présenté pour deux épisodes d'hématémèse de moyenne abondance sans mélénas associées à des douleurs chroniques de l'hypochondre droit en coups de poignard d'intensité modérée d’évolution intermittentes depuis 6 mois avec un ictère d'allure cholestatique intermittent, sans trouble de transit ni distension abdominale;le tout évoluant dans un contexte d'asthénie d'amaigrissement non chiffré et de sensation fébrile. L'examen clinique avait objectivé une pâleurcutanéo- muqueuse avec état hémodynamique stable et une sensibilité de l'hypochondre droit sans signe de Murphy ni signes d'insuffisance hépatocellulaire ni d'hypertension portale, sans masse palpable.

**Figure 1:**
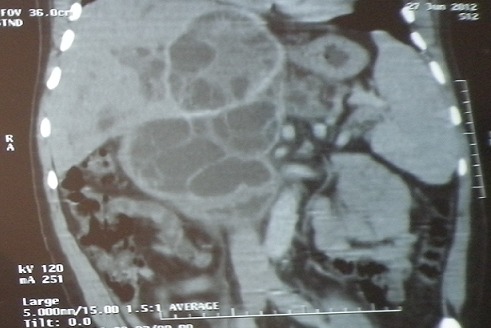
Coupe scannogaraphique frontale correspondant au kyste hydatique hépatique

**Figure 2:**
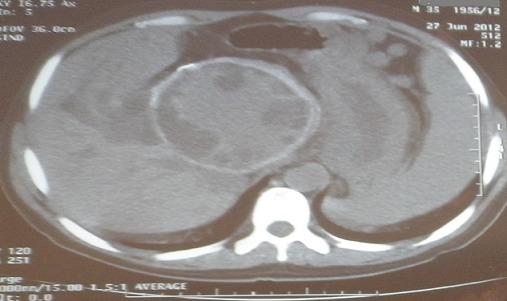
Coupe scannogaraphique frontale correspondant au kyste hydatique hépatique

Le bilan a montré: une anémie ferriprive à 10,2 g/dl, une thrombopénie à 90000/mm3, les globules blancs à 4490/mm3 avec une fonction rénale normale et un taux de prothrombine à 100%. Les transaminases étaient normales, avec une cholestase biologique minime. La fibroscopie œsogastroduodénale (FOGD) avait objectivé des VO grade III avec des varices œsogastriques type 1 et signes rouges, qui ont nécessité une ligature élastique hémostatique sans incidents. L’échographie abdominale complétée par la tomodensitométrie abdominale avaient mis en évidence un volumineux kyste hydatique de type III (Figure 1, Figure 2); interhépatogastriquedu segment I et du foie gauche, adhérent au duodénopancréas et comprimant le pédicule hépatique avec HTP secondaire et dilatation des voies biliaires intra hépatiques. La sérologie hydatique était positive. Et les sérologies virales B et C étaient négatives.

Le patient a été opéré pour son kyste hydatique. L'exploration avait retrouvé deux kystes hydatiques du foie communicant de types III; le premier sur la face inférieure du foie au niveau du petit épiploon très adhérant à la petite courbure de l'estomac et un second kyste hydatique siégeant au niveau de l'arrière cavité des épiploons et qui descend en postérieure du mésocolon transverse avec plusieurs circulations collatérales qui enveloppent les kystes hydatiques et une splénomégalie. Le geste avait consisté en une résection du dôme saillant des deux kystes hydatiques. Le patient a été mis sous albendazole avant et après la chirurgie.

L’évolution a été marquée par l'absence de récidive hémorragique avec un recul d′un an et demi.

## Discussion

Le kyste hydatique du foieest une affection parasitaire due au développement intra hépatique de l'ecchinococusgranulosus [5]. Elle demeure fréquente et constitue un problème de santé publique dans les pays de forte endémie comme: le pourtour du bassin méditerranéen, l'Afrique du nord [6]

Dans de rare cas, le KHF est révélé par une complication vasculaire comme l'HTP illustrée dans notre cas [7], cette complication est expliquée par la compression des structures vasculaires intra ou péri-hépatique (TP, ses branches, VCI, VSH) sous l'effet de la pression transmise par le kyste au coursde son expansion en l'absence de traitement [7]. A notre connaissance une dizaine de cas révélés par une hypertension portale sont décrit dans la littérature depuis la publication du premier cas dans les années 1970 [1, 3]

La compression est le plus souvent asymptomatique et tolérée. La présentation clinique est variable et dépend essentiellement de la taille du KHF et de sa localisation [8]. Il est exceptionnellement révélé par une hémorragie digestive par rupture de varice œsophagienne comme rapporté dans la littérature [8, 9], cette complication est souvent associée àune compression des voies biliaires. L'insuffisance hépatocellulaire est en général peu prononcée, mais elle dépend surtout de la durée d’évolution et de la levée ou non de l'obstruction bilioportale. La FOGD permet de faire le diagnostic des varices œsophagiennes ou gastriques et de faire un geste hémostatique endoscopique en cas de saignement en attendant le traitement étiologique qui repose sur la levée de l′obstacle par la chirurgie. Une classification de l'hypertension portale d'origine hydatique a été proposée par bourgeon et al. dans les années 80, elle comporte 4 stades [7] mais elle n'est pas utilisée.

Le diagnostic repose sur l'imagerie: L’échographie couplée au doppler est l'examen de choix [6]; elle permet de poser le diagnostic de KH dans la plupart des cas, en précisant le siège, le contenu des kystes, leur nombre ainsi que les rapports vasculaires et biliaires intra-hépatiques et d’établir la classification de Gharbi [8]. L’écho-doppler permet d’évaluer les rapports du kyste hydatique avec lesaxes vasculaires (veine porte, veines sushépatiques, veine cave inférieure) et d'objectiver selon le niveau de compression des signes directs ou indirects d'hypertension portale, parfois un cavernome porte ou un syndrome de buddchiari [7]. Le scanner: n'est pas indispensable au diagnostic, mais il est indiqué en cas de difficultés diagnostiques à l’échographie, surtout en cas de gros kystes centrauxou des kystes de type IV [6]. L'imagerie par résonance magnétique ne semble pas apporter d'avantage [5]. La sérologie hydatique est inconstamment positive [5]

Le traitement est médicochirurgical, son objectif est de lever l'obstacle représenté par le kyste hydatiquepar résection du dôme saillant ou périkystectomie. Le geste chirurgical doit être entouré par la prise de l'albendazole à commencer quatre jours avant le geste et à continuer jusqu’à quatre semaines après pour éviter la dissémination hydatique et la récidive [9, 10]. Son pronostic reste bon, mais la réversibilité de l'hypertension portale après traitement du kyste hydatique reste à prouver.

## Conclusion

La maladie hydatique reste fréquente et bénigne, cependant les complicationsvasculaires sont rares et graves. L’échographie couplée au scanner joue unrôle important dans le diagnostic de ces complications vasculaires (en montrant leur point d'impact et en établissant une cartographie vasculaire fonctionnelle précise). L'hypertension portale due à un kyste hydatique du foie semble avoir un meilleur pronostic selon les cas rapportés dans la littérature. Cetteétiologie reste néanmoins très rare et nécessite une prise en charge particulière.

## References

[1] Papadimitriou J, Kannas D, Papadimitriou L (1990). Portal hypertension due to hydatid liver disease. J R Soc Med..

[2] Klein C, Reikowski H, Müting D, Matzander U (1971). Case report on the clinical picture of hepatic echinococcosis with special reference to the occurrence of portal hypertension with bleeding esophageal varices. Med Welt..

[3] Bustíos SC, Uribe MR, Vargas CG, Myurí BC (1999). Hepatichydatic cyst associated with portal hypertension. RevGastroenterolPeru..

[4] Maamouria N, Ben Hariz F, Belkahla N, Guellouz S (2011). Syndrome de Budd-chiari: complication rare du kyste hydatique du foie: à propos de trois cas. J Afri d'Hépato-Gastroentérologie..

[5] Blairon L, Derbe F, Ben Hadj Hamida R, DelmCée M (2000). Le kyste hydatique du foie: Approche clinique et thérapeutique: A propos de 97 cas dans un CHU de Tunisie centrale. Med mal Infect..

[6] Sakhri J, Ben Ali A (2004). Le kyste hydatique du foie. J Chir..

[7] Sahnoun D, Chabchoub H, Mnif Z, Ghariani R (2006). Les complications vasculaires des kystes hydatiques du foie. Journal de Radiologie..

[8] Lahmidani N, Aqodad N, Benajah D, El Abkari M (2011). Hématémèse révélant une hypertension portale sur kyste hydatique du foie: À propos d'un cas avec revue de la littérature. J Afr Hépatol Gastroentérol..

[9] Gharbi HA, Hassine W, Brauner MW, Dupuch K (1981). Ultrasound examinationof hydaticliver. Radiology..

[10] García-Díaz JD, Ramos Ramos JC (2001). Portal hypertension ascomplication of hepatic hydatidosis. An Med Interna..

